# Quantitative Imaging Biomarkers in Age-Related Macular Degeneration and Diabetic Eye Disease: A Step Closer to Precision Medicine

**DOI:** 10.3390/jpm11111161

**Published:** 2021-11-08

**Authors:** Gagan Kalra, Sudeshna Sil Kar, Duriye Damla Sevgi, Anant Madabhushi, Sunil K. Srivastava, Justis P. Ehlers

**Affiliations:** 1 Cole Eye Institute, Cleveland Clinic, Cleveland, OH 44195, USA; kalrag2@ccf.org (G.K.); ddamlasevgi@gmail.com (D.D.S.); srivass2@ccf.org (S.K.S.); 2 Tony and Leona Campane Center for Excellence in Image-Guided Surgery & Advanced, Cleveland Clinic, Cleveland, OH 44195, USA; SXS2440@case.edu; 3 Department of Biomedical Engineering, Case Western Reserve University, Cleveland, OH 44106, USA; axm788@case.edu; 4 Louis Stokes Cleveland Veterans Administration Medical Center, Cleveland, OH 44106, USA

**Keywords:** retinal imaging, quantitative biomarkers, diabetic retinopathy, diabetic macular edema, age-related macular degeneration, precision medicine, anti-VEGF therapy

## Abstract

The management of retinal diseases relies heavily on digital imaging data, including optical coherence tomography (OCT) and fluorescein angiography (FA). Targeted feature extraction and the objective quantification of features provide important opportunities in biomarker discovery, disease burden assessment, and predicting treatment response. Additional important advantages include increased objectivity in interpretation, longitudinal tracking, and ability to incorporate computational models to create automated diagnostic and clinical decision support systems. Advances in computational technology, including deep learning and radiomics, open new doors for developing an imaging phenotype that may provide in-depth personalized disease characterization and enhance opportunities in precision medicine. In this review, we summarize current quantitative and radiomic imaging biomarkers described in the literature for age-related macular degeneration and diabetic eye disease using imaging modalities such as OCT, FA, and OCT angiography (OCTA). Various approaches used to identify and extract these biomarkers that utilize artificial intelligence and deep learning are also summarized in this review. These quantifiable biomarkers and automated approaches have unleashed new frontiers of personalized medicine where treatments are tailored, based on patient-specific longitudinally trackable biomarkers, and response monitoring can be achieved with a high degree of accuracy.

## 1. Introduction

Ophthalmology and the field of retinal diseases relies heavily on information derived from ophthalmic imaging for diagnosis, treatment and disease activity monitoring. The development of different imaging modalities, including optical coherence tomography (OCT) and ultra-widefield fluorescein angiography (UWFA), have provided incredible visualization of retinal microstructures and abnormalities, which has helped to build new insights for the management of retinal diseases, including diabetic eye disease (diabetic retinopathy, DR; diabetic macular edema, DME) and age-related macular degeneration [1,2].

Optical coherence tomography (OCT) is a rapid, non-invasive diagnostic test that provides outstanding visualization of cross-sectional and 3D morphological characteristics in addition to high-definition anatomy. OCT has become the backbone for the diagnosis and management of retinal diseases, with more than 30 million OCT scans being performed annually [3,4,5]. Due to its widespread utilization for retinal disease, OCT has become a key source for the exploration of imaging biomarkers through computational and deep learning techniques. The assessment of targeted features, such as retinal compartment volumes or volumetric pathology characterization, has been shown to be associated with disease burden and has the potential to enhance personalized treatment decisions [6,7,8]. OCT angiography (OCTA) uses non-invasive OCT technology to obtain vascular structural information by assessing decorrelation in the OCT signal due to vascular flow.

There are some important limitations to consider related to current OCT technology. With a limited field of view in most widely available OCT devices, the primary imaging location is the macula, and peripheral changes may be missed, especially in early disease [9,10]. Further, artifacts due to inconsistent montage techniques, motion-blur, and projection shadows may impact interpretation [9,10].

UWFA is an emerging imaging technique which enables visualization of panretinal vascular abnormalities including leakage, microaneurysms, and nonperfusion [2]. UWFA is a critical tool in the panretinal evaluation of retinal vascular and inflammatory disorders. With up to a 200-degree field of view, this imaging modality is the gold standard for detecting peripheral disease, especially early on in the disease process [11,12,13,14]. However, the technique does require the intravenous injection of fluorescein dye, which poses potential systemic risks [11,12,13,14]. Additionally, peripheral shadowing, eyelash artifacts, and image quality control can limit the consistency of interpretations [11,12,13].

Optical coherence tomography angiography (OCTA) is a major leap forward in this regard as it is completely non-invasive and provides high-resolution 3D binarized vessel maps that are objective and easy to interpret. The depth-encoded nature of the OCTA vascular data provides a unique advantage for evaluating the location of vascular abnormalities. However, current technology is primarily limited to macular pathology and can be subject to significant quality challenges, such as motion artifacts [11,12,14]. Additionally, OCTA does not provide information on leakage.

Current imaging systems provide outstanding details of disease burdens and the impact of different retinal diseases. Traditionally, this information has been utilized in a qualitative manner and relies on an ophthalmologist’s interpretation and expertise. This inherently introduces bias and subjectivity in the assessment of these images, and therefore may limit consistency and the opportunities for precision medicine. Additionally, all of these images encode incredible amounts of data related to the underlying imaging phenotype of a given disease. These features, such as the location and type of leakage in UWFA or the reflectivity features of cysts on OCT, may carry critical information regarding the underlying pathophysiology and driving cellular pathways of a given disease [15,16,17,18,19].

Recently, machine learning (ML) based algorithms has gained traction for use in several medical image processing operations such as organ segmentation [20], cancer detection [21] and numerous diseases including diabetic eye diseases [20,22,23]. Deep learning (DL) is a subfield of ML and uses multi-layered neural-network structures. Most typical ML models employ pre-defined or engineered features, while DL models can learn useful representations of data and features directly from the raw data itself [20,23]. Hence DL approaches are also referred to as unsupervised feature generation-based ML approaches. The opportunities for the application of DL for different ophthalmologic diseases is quite rich. DL models are not without their challenges. The opacity of DL models creates unique issues in transparency of understanding the underpinnings of classification and model performance. DL models consider segmentation or classification problem as a binary problem and does not evaluate the heterogeneity within the tissue. Optimization of the deep neural network hyperparameters is a significant challenge. The search space for the model parameters is generally very high. Also in a data scarce environment, DL models tend to perform only marginally better than random guessing [24]. 

Radiomics is an emerging field of medical image processing that refers to the computerized data extraction from medical images and aims to capture the subvisual image attributes that may not be identified by the human experts. It provides opportunity to physicians to interpret images better regarding individualized therapy, surveillance, diagnosis, and prognosis [25]. These advanced image analysis techniques have been described broadly in the domain of brain tumor [26], breast cancer [27], prostate cancer [28] and several other diseases. The role of radiomics features in predicting therapeutic response and prognosis in ophthalmic diseases is emerging as an exciting opportunity for enhanced personalized care [17,18]. 

**Figure 1:**
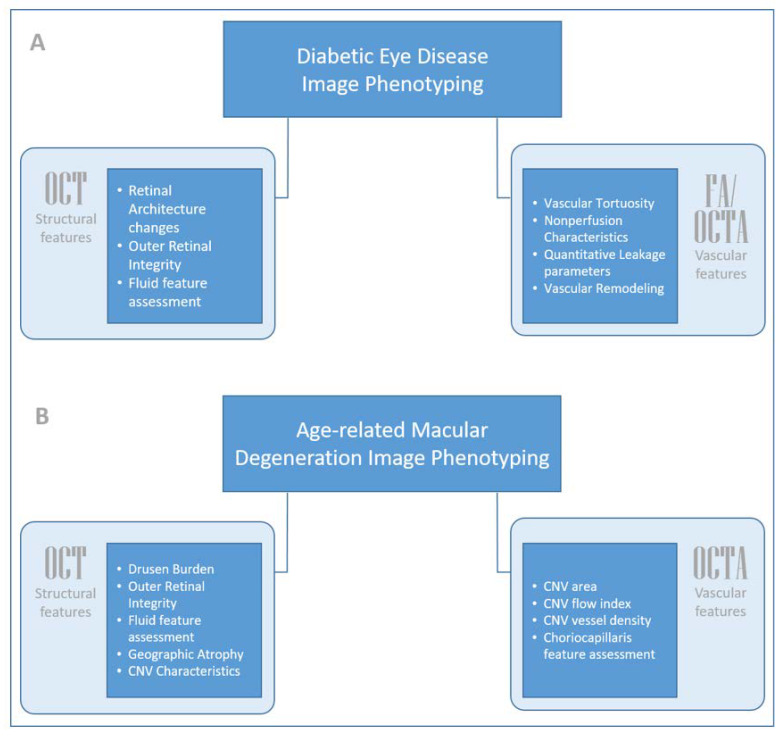
Schematic summarizing changes in various quantitative imaging biomarkers in (**A**) diabetic eye disease and (**B**) age-related macular degeneration. CNV: choroidal neovascularization.

The boom in this image analysis space over the past decade has made it possible to automate the quantification and interpretation of ophthalmic imaging biomarkers. These computational imaging elements can then be evaluated for their role as biomarkers for disease diagnosis, prognosis, treatment initiation, and therapeutic response. For this review, we describe these measured features that are found to have clinical applications for the management of disease as “quantitative imaging biomarkers”, which may serve as objective tools for the future in the context of diabetic eye disease and age-related macular degeneration (Figure 1).

***Review Methodology.*** A literature search was performed using the key words “quantitative imaging”, “diabetic retinopathy”, “age related macular degeneration”, “OCT”, “OCTA”, “fluorescein angiography”, and “quantitative biomarkers” on databases, including PubMed Central and Google Scholar. Studies reporting quantitative imaging biomarkers using OCT, OCTA, and FA in diabetic eye disease and age-related macular degeneration. Studies that included only qualitative findings or that focused on pathologies other than diabetic eye disease and AMD were not included in this study.

## 2. Diabetic Eye Disease: Diabetic Retinopathy and Diabetic Macular Edema

### 2.1. Structural Biomarkers: Optical Coherence Tomography (OCT)

#### 2.1.1. Characterizing Disease Burden and Functional Significance

**Figure 2:**
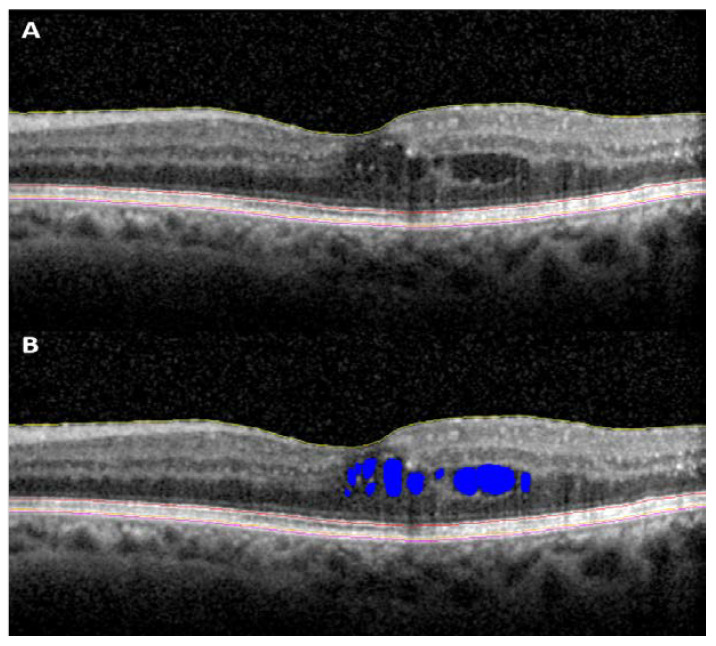
Spectral domain OCT scan of a patient with diabetic macular edema. (**A**) B-scan showing segmented retinal layers and (**B**) B-scan showing segmented retinal layers and intra-retinal fluid (shown in blue). OCT: optical coherence tomography.

In diabetic retinopathy, increased central subfield retinal thickness (CST) and de-creased retinal nerve fiber layer thickness have been associated with increased severity of retinopathy (DR) [29,30,31]. Furthermore, disruption of retinal inner layers (i.e., DRIL) has been shown to be associated with worse visual acuity in DR patients [32,33]. The presence of DRIL has been shown to have very high specificity for macular nonperfusion in DR [34]. DRIL, as well as outer retinal disruption (e.g., ellipsoid zone and external limiting membrane loss), have been shown to be associated with visual acuity in both DR and diabetic macular edema (DME) (Figure 2) [33,35]. Morphological signs such as hyperreflective foci (HRF) have been described in diabetic retinopathy and diabetic macular edema as a sign of lipid extravasation and inflammatory cellular aggregates [36,37,38]. They often initially appear in the inner retina adjacent to the native microglia, only appearing in the outer retina in more advanced stages of the disease [38]. These HRF have been shown to be aggregated activated microglial cells with numbers significantly higher in diabetic eyes when compared to controls [39,40]. HRF count has been explored as a potential biomarker to assess inflammation in diabetic eye disease. Manual and automated approaches of the segmentation of these HRF have been tested [40,41,42]. A recent study monitored the HRF counts in diabetic retinopathy and diabetic macular edema in eyes that received anti-VEGF and steroid injections. This study reported a decrease in the number of HRF with either treatment, but a more marked decrease in the steroid group [42]. This biomarker provides an interesting avenue to monitor inflammatory activity in diabetic eye disease.

Th extraction of quantitative fluid features and the assessment of retinal multi-layer segmentation has provided insights into disease prognosis and overall longitudinal disease dynamics. A recent study confirmed quantitative improvement in ellipsoid zone integrity subsequent to anti-VEGF therapy for DME [1]. This measurable improvement in ellipsoid zone integrity correlated significantly with visual function recovery. Novel higher-order imaging biomarkers, such as the retinal fluid index (RFI), are continuing to be discovered, which may help in the precise monitoring of treatment response [1,42]. Recent studies have shown that RFI volatility in the early follow-up period is correlated significantly with instability in long-term visual response to treatment [43].

#### 2.1.2. Imaging Biomarkers and Disease Pathway Expression

Utilizing these techniques, various imaging biomarkers may be able to be linked to the underlying pathways involved in disease pathogenesis. In a recent study assessing quantitative imaging biomarkers and cytokine expression, the levels of multiple cytokines (e.g., vascular endothelial growth factor (VEGF), monocyte chemotactic protein-1 (MCP-1), and interleukin-6 (IL-6)) were linked with various imaging biomarkers, such as fluid parameters and outer retinal integrity [15]. The identification of these critical components of imaging phenotype and cytokine expression may help to identify eyes that may tolerate longer intervals in-between treatments or eyes that may benefit from emerging therapeutics with novel targets.

#### 2.1.3. Predicting Future Treatment Need and Treatment Response Characteristics

Utilizing an attention-based convolutional neural network (CNN) model using pre-treatment OCT scans that preserved and highlighted the global structures in OCT images and enhanced local features from fluid/exudate-affected regions, Rasti et al. utilized retinal thickness information for the prediction of the response to intravitreal anti-VEGF treatment [44]. An additional DL algorithm developed by Prahs et al. distinguished retinal OCT B-scans that required an intravitreal injection from those that did not [45].

Beyond evaluating for treatment need, additional studies have assessed specific retinal compartment radiomics features that may predict therapeutic response. In a recent study [18], the relevance of radiomics features extracted from different spatial compartments of the retina on OCT scans to identify the patients with DME who tolerate an extension in the intervals between treatment with anti-VEGF treatment were evaluated. Texture-based radiomic features within the intraretinal fluid subcompartment were found to be most associated with a response to anti-VEGF therapy and most strongly associated in discriminating rebounders from the non-rebounders of anti-VEGF treatment following treatment interval extension.

### 2.2. Vascular Biomarkers: Ultra-Widefield Fluorescein Angiography (UWFA)

**Figure 3:**
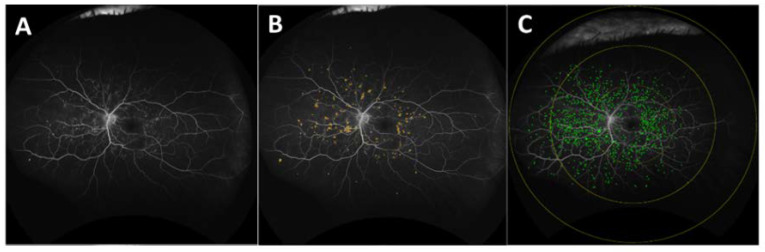
(**A**) Dewarped late-phase UWFA scan; (**B**) dewarped late-phase UWFA scan with overlay (yellow) indicating the area occupied by leakage; (**C**) dewarped early phase UWFA scan with overlay (green) indicating individual MAs in 3 mm (macular), 6 mm, and 9 mm zones (yellow circles). UWFA: ultra-widefield fluorescein angiography.

Ultra-widefield fluorescein angiography (UWFA) can capture 200° field of view (FOV) compared to conventional imaging with 30–60° FOV, enabling a more comprehensive disease evaluation. [46,47] Visualization of specific vascular features that enhance assessment of disease burden and optimize diagnostic accuracy make this modality an essential tool for the evaluation of posterior segment disorders. Areas of nonperfusion, vascular leakage, microaneurysm count, and neovascularization are among known clinically apparent biomarkers that assist diagnosis, choice of treatment and assessment of treatment response. Emerging image analysis methods provide the opportunity for manual and automated quantification of known angiographic features and discovery of novel and more complex features. The labor-intensive nature of manual feature assessment is a barrier to more widespread use. Recently, methods and systems have been developed to provide in-depth evaluation of leakage features, microaneurysm counts, ischemic burden and vascular characteristics (Figure 3) [48,49]. Machine learning systems have provided the ability to perform enhanced vascular segmentation, feature extraction, and more efficient methods for evaluating imaging characteristics [50,51,52].

#### 2.2.1. Biomarkers for Disease Severity and Disease Burden

Various biomarkers are investigated for severity grading, progression, and treatment response. Nonperfusion area, ischemic index, leakage, and microaneurysm counts have been shown to correlate strongly with the clinical severity of DR and treatment response [53]. Ehlers et al. demonstrated quantitative UWFA parameters, including panretinal MA count, ischemia, and leakage index, that were strongly associated with DR severity in 339 eyes [54]. Assessment of these disease burden metrics may help in predicting the risk of progression or DR-related complications. The panretinal leakage index has shown promise as a potential predictor of disease-related complications, such as vitreous hemorrhage and DME [53,55]. Quantification of these features allows for the longitudinal tracking of numerical changes that can be used to guide clinical decisions and assess response to treatment.

The spatial distribution of DR lesions on ultra-widefield photography including MA, cotton wool spots, intraretinal microvascular abnormalities, neovascularization, and fibrovascular proliferation was investigated in a large study with 1406 eyes demonstrating a predominantly central distribution in 63% of eyes [56]. Silva et al.’s study on nonperfusion distribution reported higher DR severity in eyes with predominantly peripheral lesions [57].

#### 2.2.2. Evaluating and Predicting Treatment Response Characteristics

In addition to the assessment of disease burden, quantitative feature characterization can also be used to assess treatment response. In an automated UWFA approach, intravitreal anti-VEGF therapy demonstrated significant and stark improvements in leakage index and total microaneurysm counts in DR [55,58]. Wykoff et al. reported that the ischemic index increased by 34% in one year with quarterly aflibercept (*p* = 0.009) and 10% in monthly aflibercept (*p* = 0.18) treatment [59]. In a prospective clinical trial, the authors studied the change in the panretinal leakage index in eyes with DME with aflibercept therapy to quantify therapeutic response. The authors noted a dramatic reduction in the leakage index (from 3.5% at baseline to 0.4% at 12 months) with aflibercept therapy [58]. Utilizing quantitative UWFA in the RECOVERY study, which evaluated eyes with severe PDR, quantitative UWFA demonstrated a dramatic reduction of 68% to 79% in leakage index reduction at 1 year, with similar outcomes in both monthly and quarterly dosing [55]. In a randomized controlled trial comparing leakage-index-guided treatment and Diabetic Retinopathy Severity Scale (DRSS)-level-guided treatment with intravitreal aflibercept for DR, the authors found that deteriorations in the leakage index preceded those in the DRSS level, thereby providing a potentially higher sensitivity marker for the need for retreatment [60].

#### 2.2.3. Imaging Biomarkers and Disease Pathway Expression

Another recent study assessed the correlation between UWFA imaging phenotype and cytokine expression in eyes with DME from the IMAGINE study [16]. The authors noted that an increased panretinal leakage index correlated strongly with VEGF, angiopoietin-like 4, and interleukin-6 levels, while the panretinal ischemic index was positively correlated with the tissue inhibitor of metalloproteinases 1 (TIMP-1) and VEGF [16]. Further research is needed to understand the implications of these phenotype–cytokine expression correlations in assessing response to treatment.

#### 2.2.4. Radiomics Angiographic Biomarkers for DR Severity

In addition to clinically apparent biomarkers, Fan et al. demonstrated the branching complexity of peripheral vessels and the distribution of nonperfusion areas correlated with DR severity [61]. Fractal dimension (FD) depicts the complexity of vascular geometry, such that higher values indicate dense, intricate, space-filling branching patterns [62]. Peripheral retinal vessels of diabetic eyes have been demonstrated to have lower complexity in their branching pattern (fractal dimension) compared to healthy controls. FD was shown to be negatively associated with the nonperfusion area [63]. A significantly lower FD is noted in the retinal vasculature in DR, especially in the far peripheral fields when compared to normal eyes. Additionally, a decrease in panretinal FD was shown to be associated with an increase in the global nonperfusion area [64]. In addition to FD, the skewness of retinal vasculature density distribution has also been associated with DR severity [65].

#### 2.2.5. Angiographic Biomarkers for DME Presence

Quantitative UWFA has also been explored in DME pathogenesis. The leakage index and MA count in the posterior pole have been associated with the presence and severity of DME [53]. The nonperfusion distribution pattern in DR was observed in DME, being more extensive in mid-periphery ischemia compared to the posterior pole and far periphery. Fang et al. classified ischemic areas and investigated nonperfusion with and without leakage in DME eyes [66]. Nonperfusion areas with leakage were found more extensively in the posterior retina compared to nonperfusion without leakage, which is predominantly in the mid-periphery [66]. A nonperfusion area with leakage positively correlated whereas nonperfusion without leakage negatively correlated with DME severity.

#### 2.2.6. Evaluating and Predicting Therapeutic Response: From Quantitative UWFA to Radiomics

Quantitative UWFA biomarkers have been explored as assessment tools for therapeutic response in eyes with DME treated with aflibercept in the PERMEATE study [58]. Aflibercept injections resulted in a 78% decrease in the leakage index of eyes with DME. Similar to the outcome in eyes with DR, the nonperfusion area is increased despite anti-VEGF therapy [58].

Beyond characterizing the longitudinal quantitative UWFA feature alterations in response to therapy, radiomics features have been utilized to predict treatment response and durability. Prasanna et al. developed novel radiomic CIBs that characterized different morphological properties of leakage nodes and vascular tortuosity on UWFA, which were linked to the durability of anti-VEGF treatment [17]. The distribution of leakage nodes in eyes that did not tolerate treatment extension was found to be more disordered than eyes that tolerated an extension in the intervals between treatment. Vessel tortuosity was increased and more complex in eyes that experienced clinical worsening following treatment extension. In a supportive assessment of radiomics features for predicting treatment response characteristics, Moosavi et al. identified that the proximity of leakage foci to the vessels has a higher variance in eyes who have more durable treatment response, whereas increased local vascular tortuosity was linked to reduction in tolerance of treatment extension [67].

### 2.3. Vascular Biomarkers: OCTA

OCTA uses non-invasive OCT technology to obtain vascular structural information by assessing decorrelation in the OCT signal due to vascular flow. As a result of the depth resolution of OCTA, different chorioretinal vascular plexuses, such as the nerve fiber layer plexus (NFLP), ganglion cell layer plexus (GCLP), intermediate capillary plexus (ICP) and deep capillary plexus (DCP), have been studied using this technology. NFLP and GCLP form the superficial vascular complex while ICP and DCP form the deep vascular complex [68].

#### Biomarkers for Disease Severity and Burden: From Quantitative Features to Radiomics

An increased foveal avascular zone (FAZ) size is noted in patients with DR compared to normal [69,70,71]. Recent OCTA studies have provided evidence for a correlation between FAZ size and visual acuity, such that an increase in FAZ size is associated with decreased visual acuity [72,73,74]. In addition to FAZ area, the shape of the FAZ has been shown to change in various DR grades [75].

Vessel density, as calculated from OCTA, has been shown to be inversely correlated with DR grade in multiple trials [70,76,77]. In a study characterizing the association between visual acuity and vessel density in DR, vessel density was reduced in eyes with decreased visual acuity [78].

Vessel diameter index (VDI) is a representation of vessel diameter obtained by calculating a ratio of the total area of the scan occupied by blood vessels and the total skeletonized length of blood vessels in the scan. In a recent study, the VDI obtained using OCTA has been shown to positively correlate with the severity of DR and blood glucose levels [79,80,81].

Similar to UWFA, retinal vessel tortuosity in OCTA is another important metric that holds high potential for the evaluation of DR. Vascular tortuosity on OCTA positively correlates with the severity of DR in superficial and deep retinal vascular plexuses in moderate to severe DR [75]. In a recent study, vessel tortuosity demonstrated a positive correlation with DR severity in NPDR, but decreased significantly in PDR [75]. Recently, three-dimensional volume-rendering biomarkers such as vessel sphericity and cylindricity were used to assess blood vessel shape, demonstrating potential differences between normal eyes and eyes with DR [82]. Geometric features, such as vessel branching angle and vessel-width-based features have also been noted to be significantly different between normal eyes and eyes with DR [83].

## 3. Age-Related Macular Degeneration (AMD): Neovascular and Non-Neovascular AMD

### 3.1. Structural Biomarkers: Optical Coherence Tomography (OCT)

#### 3.1.1. Features for Predicting Progression in AMD

Non-neovascular (i.e., dry) age-related macular degeneration has been extensively evaluated for numerous imaging biomarkers such as intraretinal hyper-reflective foci (HRF), complex drusenoid lesions (DL, i.e., heterogeneous reflectivity), subretinal drusenoid deposits (SDDs), and drusen burden. SD-OCT has been used to qualitatively describe these biomarkers and has confirmed that each of these features confers a greater risk of disease progress [84,85]. In a recent study, quantitative EZ integrity measures, EZ mapping, and sub-RPE compartment quantification were shown to be important predictors of progression to geographic atrophy in nonexudative AMD patients [86]. Specifically, the reduced EZ integrity and increased sub-RPE compartment thickness was identified in eyes that progressed to subfoveal geographic atrophy. These quantitative biomarkers were more strongly associated with progression than qualitative features, such as HRF and SDD. Utilizing a ML classifier, a high-performance system was developed for predicting progression to subfoveal GA based on multiple quantitative outer retinal features [87,88].

Automated drusen volume quantification has been enabled by multi-layer segmentation platforms that provide isolation of the sub-RPE compartment. One study demonstrated that an increase in the drusen volume was associated with a significant increase in the risk of developing geographic atrophy or conversion to neovascular AMD [89]. ML-enhanced systems for advanced segmentation and feature extraction are creating new opportunities for automated disease characterization and longitudinal monitoring of therapeutic response in AMD. Multiple studies have demonstrated volumetric fluid characterization, compartment-specific OCT feature evaluation (such as ellipsoid zone integrity), and volumetric quantification of subretinal fibrosis as well as subretinal hyperreflective material [6,90,91]. In a recent study utilizing deep learning for the extraction of quantitative features in AMD patients, the authors noted that an increase in drusen volume, SRF, IRF, serous pigment epithelium detachment, HRF and subretinal hyperreflective material was associated with worse visual acuity [7].

#### 3.1.2. Deep Learning and Radiomics Biomarkers in AMD 

DL-based analysis systems have been explored to detect the presence of disease. Multiple other studies have shown the effectiveness of DL models in classifying normal versus AMD eyes from OCT images [92,93]. Automated SD-OCT image analysis using DL techniques are currently widely used for predicting disease progression in AMD. Predicting conversion from early or intermediate non-neovascular AMD to neovascular AMD using quantitative imaging features (e.g., drusen shape, drusen volume) in SD-OCT images has been previously explored [94,95]. Banerjee et al. proposed a hybrid sequential model integrating hand-crafted size-based and shape-based radiomics features (related to the relationship of image intensity between voxels), demographic and visual acuity data, and DL with a recursive neural network (RNN) model in the same platform to predict the probability of future neovascular conversion [22].

### 3.2. Vascular Biomarkers: OCTA

In neovascular AMD, CNV is a major cause of vision loss due to photoreceptor damage that results from exudation processes [96,97]. Although FA has traditionally been the gold standard to characterize and identify CNV lesions, OCT has now become the benchmark evaluation for the presence of CNV and exudation. OCTA is also emerging as a promising technology for the high-level visualization of neovascular membranes in neovascular AMD and for evaluating the choriocapillaris in non-neovascular AMD [98,99,100].

#### 3.2.1. Quantitative Biomarkers of CNV Features

In one study aimed at characterizing CNV using quantitative biomarkers on OCTA, the CNV area and flow index using outer retinal choriocapillaris OCTA slabs for assessment of CNV characterization [101]. The study identified a higher flow in larger CNVs and those that were type II [101]. In a recent study, the quantification of CNV and other vascular characteristics was evaluated to assess treatment response to anti-VEGF therapy in neovascular AMD patients [102]. Eyes requiring more frequent dosing of anti-VEGF agents had lower CNV vessel density compared to groups with longer duration intervals between doses [102]. Further, the CNV area was noted to be higher in eyes with fovea involvement and core vessel presence. Absence of these findings may therefore be suggestive of inactive CNV.

#### 3.2.2. Choriocapillaris Biomarkers in Non-Neovascular AMD

In non-neovascular AMD, OCTA has been explored to study many aspects of the disease process such as drusen, reticular pseudodrusen, and geographic atrophy, in addition to exploring its utility for the monitoring of disease progression [100]. Choriocapillaris flow depletion in eyes with drusen has been shown on OCTA [103,104]. Reduced flow may result in relative hypoxia of outer retinal layers and disease progression. In a recent study, quantitative assessment of choriocapillaris flow deficits demonstrated reduced flow in eyes with drusen with hyporeflective cores compared with eyes with drusen without hyporeflective cores, suggesting that the presence of hyporeflective cores may indeed indicate a more advanced disease process in intermediate AMD [105]. OCTA has been used to characterize geographic atrophy (GA) as well, particularly choriocapillaris flow deficits [106]. Focal perfusion loss (FPL) on OCTA has been used to evaluate choriocapillaris flow features in AMD, which has been identified to be higher in AMD eyes compared to controls [107].

## 4. Conclusions

**Figure 4:**
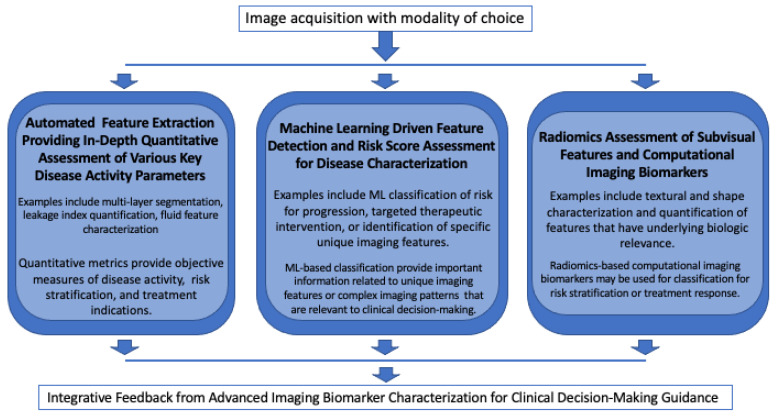
Developing the “Imaging Phenotype”. A potential multi-factorial approach for integrative imaging biomarker characterization utilizing multiple advanced feature interrogation and extraction methods.

Quantitative imaging biomarkers derived from multiple imaging modalities may provide a critical platform for the future in providing objective and trackable metrics that enable precision medicine in ophthalmic care through the comprehensive characterization of the “imaging phenotype”, Figure 4.

OCT imaging biomarkers provide valuable structural information of retinal layers, such as retinal compartment thickness, layer integrity maps, fluid volume, and the fluid index. UWFA and OCTA imaging biomarkers provide key information regarding the retinal and choroidal vasculature, such as measures of vessel density, ischemic area, flow voids, leakage area, leakage index, ischemic index, and the CNV area. Radiomics is an emerging field in ophthalmology and is having an increasingly high impact on personalized medicine. As the field matures in the future, a combination of different novel DL networks and advanced radiomic methods may be of high value for a more complete decision support system (Figure 4). The implementation of deep learning, advanced feature interrogation methods, and radiomics characterization provides an exciting opportunity for enhanced understanding of and new insights into retinal disease. The field of computational imaging biomarker discovery and exploration in AMD and diabetic eye disease is emerging as a major opportunity for personalized care and precision medicine.

## References

[1] Ehlers J.P., Uchida A., Hu M., Figueiredo N., Kaiser P.K., Heier J.S., Brown D.M., Boyer D.S., Do D.V., Gibson A. (2019). Higher-Order Assessment of OCT in Diabetic Macular Edema from the VISTA Study: Ellipsoid Zone Dynamics and the Retinal Fluid Index. Ophthalmol. Retin..

[2] Querques L., Parravano M., Sacconi R., Rabiolo A., Bandello F., Querques G. (2017). Ischemic index changes in diabetic retinopathy after intravitreal dexamethasone implant using ultra-widefield fluorescein angiography: A pilot study. Acta Diabetol..

[3] Bourne R.R., Flaxman S.R., Braithwaite T., Cicinelli M.V., Das A., Jonas J.B., Keeffe J., Kempen J.H., Leasher J., Limburg H. (2017). Magnitude, temporal trends, and projections of the global prevalence of blindness and distance and near vision impairment: A systematic review and meta-analysis. Lancet Glob. Health.

[4] Kurmann T., Yu S., Márquez-Neila P., Ebneter A., Zinkernagel M.S., Munk M.R., Wolf S., Sznitman R. (2019). Expert-level Automated Biomarker Identification in Optical Coherence Tomography Scans. Sci. Rep..

[5] Schmidt-Erfurth U., Klimscha S., Waldstein S.M., Bogunović H. (2016). A view of the current and future role of optical coherence tomography in the management of age-related macular degeneration. Eye.

[6] Ehlers J.P., Clark J., Uchida A., Figueiredo N., Babiuch A., Talcott K.E., Lunasco L., Le T.K., Meng X., Hu M. (2021). Longitudinal Higher-Order OCT Assessment of Quantitative Fluid Dynamics and the Total Retinal Fluid Index in Neovascular AMD. Transl. Vis. Sci. Technol..

[7] Moraes G., Fu D.J., Wilson M., Khalid H., Wagner S.K., Korot E., Ferraz D., Faes L., Kelly C.J., Spitz T. (2021). Quantitative Analysis of OCT for Neovascular Age-Related Macular Degeneration Using Deep Learning. Ophthalmology.

[8] Ehlers J.P., Khan M., Petkovsek D., Stiegel L., Kaiser P., Singh R.P., Reese J.L., Srivastava S.K. (2018). Outcomes of Intraoperative OCT–Assisted Epiretinal Membrane Surgery from the PIONEER Study. Ophthalmol. Retin..

[9] Reznicek L., Kolb J.P., Klein T., Mohler K.J., Wieser W., Huber R., Kernt M., Märtz J., Neubauer A.S. (2015). Wide-Field Megahertz OCT Imaging of Patients with Diabetic Retinopathy. J. Diabetes Res..

[10] De Pretto L.R., Moult E.M., Alibhai A.Y., Carrasco-Zevallos O.M., Chen S., Lee B.K., Witkin A.J., Baumal C.R., Reichel E., de Freitas A.Z. (2019). Controlling for artifacts in widefield optical coherence tomography angiography measurements of non-perfusion area. Sci. Rep..

[11] de Carlo T.E., Bonini Filho M.A., Baumal C.R., Reichel E., Rogers A., Witkin A.J., Duker J.S., Waheed N.K. (2016). Evaluation of preretinal neovascularization in proliferative diabetic retinopathy using optical coherence tomography angiography. Ophthalmic Surg. Lasers Imaging Retin..

[12] Sawada O., Ichiyama Y., Obata S., Ito Y., Kakinoki M., Sawada T., Saishin Y., Ohji M. (2018). Comparison between wide-angle OCT angiography and ultra-wide field fluorescein angiography for detecting non-perfusion areas and retinal neovascularization in eyes with diabetic retinopathy. Graefe’s Arch. Clin. Exp. Ophthalmol..

[13] Kaines A., Tsui I., Sarraf D., Schwartz S. (2009). The Use of Ultra Wide Field Fluorescein Angiography in Evaluation and Management of Uveitis. Semin. Ophthalmol..

[14] Couturier A., Rey P.-A., Erginay A., Lavia C., Bonnin S., Dupas B., Gaudric A., Tadayoni R. (2019). Widefield OCT-Angiography and Fluorescein Angiography Assessments of Nonperfusion in Diabetic Retinopathy and Edema Treated with Anti–Vascular Endothelial Growth Factor. Ophthalmology.

[15] Abraham J.R., Wykoff C.C., Arepalli S., Lunasco L., Hannah J.Y., Hu M., Reese J., Krovastava S.K., Brown D.M., Ehlers J.P. (2021). Aqueous cytokine expression and higher order OCT biomarkers: Assessment of the Anatomic-Biologic bridge in the IMAGINE DME study. Am. J. Ophthalmol..

[16] Abraham J.R., Wykoff C.C., Arepalli S., Lunasco L., Hannah J.Y., Martin A., Mugnaini C., Hu M., Reese J., Strivastava S.K. (2021). Exploring the angiographic-biologic phenotype in the IMAGINE study: Quantitative UWFA and cytokine expression. Br. J. Ophthalmol..

[17] Prasanna P., Bobba V., Figueiredo N., Sevgi D.D., Lu C., Braman N., Alilou M., Sharma S., Srivastava S.K., Madabhushi A. (2020). Radiomics-based assessment of ultra-widefield leakage patterns and vessel network architecture in the PERMEATE study: Insights into treatment durability. Br. J. Ophthalmol..

[18] Kar S.S., Sevgi D.D., Dong V., Srivastava S.K., Madabhushi A., Ehlers J.P. (2021). Multi-Compartment Spatially-Derived Radiomics From Optical Coherence Tomography Predict Anti-VEGF Treatment Durability in Macular Edema Secondary to Retinal Vascular Disease: Preliminary Findings. IEEE J. Transl. Eng. Health Med..

[19] Sil K.S., Sevji D.D., Dong V., Srivastava S.K., Madabhushi A., Ehlers J.P. (2021). Multi-Compartment OCT-derived Radiomics Features to predict Anti-VEGF Treatment Durability for Diabetic Macular Edema. Investig. Ophthalmol. Vis. Sci..

[20] Akkus Z., Galimzianova A., Hoogi A., Rubin D.L., Erickson B.J. (2017). Deep Learning for Brain MRI Segmentation: State of the Art and Future Directions. J. Digit. Imaging.

[21] Sumathipala Y., Lay N.S., Turkbey B. (2018). Prostate cancer detection from multi-institution multiparametric MRIs using deep convolutional neural networks. J. Med. Imaging.

[22] Banerjee I., De Sisternes L., Hallak J.A., Leng T., Osborne A., Rosenfeld P.J., Gregori G., Durbin M., Rubin D. (2020). Prediction of age-related macular degeneration disease using a sequential deep learning approach on longitudinal SD-OCT imaging bi-omarkers. Sci. Rep..

[23] Lundervold A., Lundervold A. (2019). An overview of deep learning in medical imaging focusing on MRI. Z. Med. Phys..

[24] Dong V., Sevgi D.D., Kar S.S., Srivastava S.K., Ehlers J.P., Madabhushi A. (2021). Evaluating the utility of deep learning using ultra-widefield fluorescein angiography for predicting need for anti-VEGF therapy in diabetic eye disease. Investig. Ophthalmol. Visual Sci..

[25] Rizzo S., Botta F., Raimondi S., Origgi D., Fanciullo C., Morganti A.G., Bellomi M. (2018). Radiomics: The facts and the challenges of image analysis. Eur. Radiol. Exp..

[26] Wu G., Chen Y., Wang Y., Yu J., Lv X., Ju X., Shi Z., Chen L., Chen Z. (2018). Sparse Representation-Based Radiomics for the Diagnosis of Brain Tumors. IEEE Trans. Med. Imaging.

[27] Parekh V.S., Jacobs M.A. (2017). Integrated radiomic framework for breast cancer and tumor biology using advanced machine learning and multiparametric MRI. NPJ Breast Cancer.

[28] Penzias G., Singanamalli A., Elliott R., Gollamudi J., Shih N., Feldman M., Stricker P., Delprado W., Tiwari S., Böhm M. (2018). Identifying the morphologic basis for radiomic features in distinguishing different Gleason grades of prostate cancer on MRI: Preliminary findings. PLoS ONE.

[29] Vujosevic S., Midena E. (2013). Retinal Layers Changes in Human Preclinical and Early Clinical Diabetic Retinopathy Support Early Retinal Neuronal and Müller Cells Alterations. J. Diabetes Res..

[30] Shi R., Guo Z., Wang F., Lin R., Zhao L. (2018). Alterations in retinal nerve fiber layer thickness in early stages of diabetic reti-nopathy and potential risk factors. Curr. Eye Res..

[31] Deák G.G., Schmidt-Erfurth U., Jampol L.M. (2018). Correlation of Central Retinal Thickness and Visual Acuity in Diabetic Macular Edema. JAMA Ophthalmol..

[32] Joltikov K., Sesi C.A., De Castro V.M., Davila J.R., Anand R., Khan S.M., Farbman N., Jackson G.R., Johnson C.A., Gardner T.W. (2018). Disorganization of Retinal Inner Layers (DRIL) and Neuroretinal Dysfunction in Early Diabetic Retinopathy. Investig. Opthalmol. Vis. Sci..

[33] Sun J.K., Lin M.M., Lammer J., Prager S., Sarangi R., Silva P.S., Aiello L.P. (2014). Disorganization of the Retinal Inner Layers as a Predictor of Visual Acuity in Eyes With Center-Involved Diabetic Macular Edema. JAMA Ophthalmol..

[34] Nicholson L., Ramu J., Triantafyllopoulou I., Patrao N.V., Comyn O., Hykin P., Sivaprasad S. (2015). Diagnostic accuracy of disorganization of the retinal inner layers in detecting macular capillary non-perfusion in diabetic retinopathy. Clin. Exp. Ophthalmol..

[35] Eliwa T.F., Hussein M.A., Zaki M.A., Raslan O.A. (2018). Outer retinal layer thickness as good visual predictor in patients with diabetic macular edema. Retina.

[36] Bolz M., Schmidt-Erfurth U., Deak G., Mylonas G., Kriechbaum K., Scholda C. (2009). Optical Coherence Tomographic Hyperreflective Foci: A Morphologic Sign of Lipid Extravasation in Diabetic Macular Edema. Ophthalmology.

[37] Vujosevic S., Bini S., Midena G., Berton M., Pilotto E., Midena E. (2013). Hyperreflective Intraretinal Spots in Diabetics without and with Nonproliferative Diabetic Retinopathy: AnIn VivoStudy Using Spectral Domain OCT. J. Diabetes Res..

[38] Lee H., Jang H., A Choi Y., Kim H.C., Chung H. (2018). Association Between Soluble CD14 in the Aqueous Humor and Hyperreflective Foci on Optical Coherence Tomography in Patients With Diabetic Macular Edema. Investig. Opthalmol. Vis. Sci..

[39] De Benedetto U., Sacconi R., Pierro L., Lattanzio R., Bandello F. (2015). Optical coherence tomographic hyperreflective foci in early stages of diabetic retinopathy. Retina.

[40] Okuwobi I.P., Ji Z., Fan W., Yuan S., Bekalo L., Chen Q. (2019). Automated Quantification of Hyperreflective Foci in SD-OCT With Diabetic Retinopathy. IEEE J. Biomed. Health Inform..

[41] Rübsam A., Wernecke L., Rau S., Pohlmann D., Müller B., Zeitz O., Joussen A.M. (2021). Behavior of SD-OCT Detectable Hyperreflective Foci in Diabetic Macular Edema Patients after Therapy with Anti-VEGF Agents and Dexamethasone Implants. J. Diabetes Res..

[42] Roberts P.K., Vogl W.D., Gerendas B.S., Glassman A.R., Bogunovic H., Jampol L.M., Schmidt-Erfurth U.M. (2020). Quantification of fluid resolution and visual acuity gain in patients with diabetic macular edema using deep learning: A post hoc analysis of a randomized clinical trial. JAMA Ophthalmol..

[43] Ehlers J.P., Uchida A., Sevgi D.D., Hu M., Reed K., Berliner A., Vitti R., Chu K., Srivastava S.K. (2021). Retinal Fluid Volatility Associated with Interval Tolerance and Visual Outcomes in Diabetic Macular Edema in the VISTA Phase III Trial. Am. J. Ophthalmol..

[44] Rasti R., Allingham M.J., Mettu P.S., Kavusi S., Govind K., Cousins S.W., Farsiu S. (2020). Deep learning-based single-shot pre-diction of differential effects of anti-VEGF treatment in patients with diabetic macular edema. Biomed. Opt. Express.

[45] Prahs P., Radeck V., Mayer C., Cvetkov Y., Cvetkova N., Helbig H., Märker D. (2018). OCT-based deep learning algorithm for the evaluation of treatment indication with anti-vascular endothelial growth factor medications. Graefe’s Arch. Clin. Exp. Ophthalmol..

[46] Manivannan A., Plskova J., Farrow A., Mckay S., Sharp P.F., Forrester J.V. (2005). Ultra-Wide-Field Fluorescein Angiography of the Ocular Fundus. Am. J. Ophthalmol..

[47] Falavarjani K.G., Wang K., Khadamy J., Sadda S.R. (2016). Ultra-wide-field imaging in diabetic retinopathy; an overview. J. Curr. Ophthalmol..

[48] Rabbani H., Allingham M.J., Mettu P.S., Cousins S.W., Farsiu S. (2015). Fully Automatic Segmentation of Fluorescein Leakage in Subjects With Diabetic Macular Edema. Investig. Opthalmol. Vis. Sci..

[49] Ehlers J.P., Wang K., Vasanji A., Hu M., Srivastava S.K. (2017). Automated quantitative characterisation of retinal vascular leakage and microaneurysms in ultra-widefield fluorescein angiography. Br. J. Ophthalmol..

[50] O’Connell M., Sevgi D.D., Srivastava S.K., Whitney J., Hach J.M., Atwood R., Springer Q., Williams J., Vasanji A., Reese J. (2020). Longitudinal precision of vasculature parameter assessment on ultra-widefield fluorescein angiography using a deep-learning model for vascular segmentation in eyes without vascular pathology. Investig. Ophthalmol. Vis. Sci..

[51] Sevgi D.D., Hach J., Srivastava S.K., Wykoff C., O’connell M., Whitney J., Reese J., Ehlers J.P. (2020). Automated quality optimized phase selection in ultra-widefield angiography using retinal vessel segmentation with deep neural networks. Investig. Ophthalmol. Vis. Sci..

[52] Sevgi D.D., Scott A.W., Martin A., Mugnaini C., Patel S., Linz M.O., Nti A., Reese J., Ehlers J.P. (2020). Longitudinal assessment of quantitative ultra-widefield ischaemic and vascular parameters in sickle cell retinopathy. Br. J. Ophthalmol..

[53] Jiang A.C., Srivastava S.K., Hu M., Figueiredo N., Babiuch A., Boss J.D., Reese J.L., Ehlers J.P. (2020). Quantitative Ultra-Widefield Angiographic Features and Associations with Diabetic Macular Edema. Ophthalmol. Retin..

[54] Ehlers J.P., Jiang A.C., Boss J.D., Hu M., Figueiredo N., Babiuch A., Talcott K., Sharma S., Hach J., Le T.K. (2019). Quantitative Ultra-Widefield Angiography and Diabetic Retinopathy Severity. Ophthalmology.

[55] Babiuch A.S., Wykoff C.C., Srivastava S.K., Talcott K., Zhou B., Hach J., Hu M., Reese J.L., Ehlers J.P. (2020). Retinal Leakage Index Dynamics On Ultra-Widefield Fluorescein Angiography In Eyes Treated With Intravitreal Aflibercept For Proliferative Diabetic Retinopathy In The Recovery Study. Retina.

[56] Verma A., Alagorie A.R., Ramasamy K., van Hemert J., Yadav N., Pappuru R.R., Tufail A., Nittala M.G., Sadda S.R., Indian Retina Research Associates (IRRA) (2020). Distribution of peripheral lesions identified by mydriatic ultra-wide field fundus imaging in diabetic retinopathy. Graefe’s Arch. Clin. Exp. Ophthalmol..

[57] Silva P.S., Cruz A.J.D., Ledesma M.G., van Hemert J., Radwan A., Cavallerano J., Aiello L.M., Sun J.K. (2015). Diabetic Retinopathy Severity and Peripheral Lesions Are Associated with Nonperfusion on Ultrawide Field Angiography. Ophthalmology.

[58] Figueiredo N., Srivastava S.K., Singh R.P., Babiuch A., Sharma S., Rachitskaya A., Talcott K., Reese J., Hu M., Ehlers J.P. (2020). Longitudinal Panretinal Leakage and Ischemic Indices in Retinal Vascular Disease after Aflibercept Therapy. Ophthalmol. Retin..

[59] Wykoff C.C., Nittala M.G., Zhou B., Fan W., Velaga S.B., Lampen S.I., Rusakevich A., Ehlers J.P., Babiuch A., Brown D.M. (2019). Intravitreal Aflibercept for Retinal Nonperfusion in Proliferative Diabetic Retinopathy. Ophthalmol. Retin..

[60] Yu H.J., Ehlers J.P., Sevgi D.D., Hach J., O’Connell M., Reese J.L., Srivastava S.K., Wykoff C.C. (2021). Real-Time Photographic- and Fluorescein Angiographic-Guided Management of Diabetic Retinopathy: Randomized PRIME Trial Outcomes. Am. J. Ophthalmol..

[61] Fan W., Nittala M.G., Velaga S.B., Hirano T., Wykoff C.C., Ip M., Lampen S.I., van Hemert J., Fleming A., Verhoek M. (2019). Distribution of Nonperfusion and Neovascularization on Ultrawide-Field Fluorescein Angiography in Proliferative Diabetic Retinopathy (RECOVERY Study): Report 1. Am. J. Ophthalmol..

[62] Mainster M.A. (1990). The fractal properties of retinal vessels: Embryological and clinical implications. Eye.

[63] Fan W., Uji A., Wang K., Falavarjani K.G., Wykoff C.C., Brown D.M., Van Hemert J., Sagong M., Sadda S.R., Ip M. (2020). Severity Of Diabetic Macular Edema Correlates With Retinal Vascular Bed Area On Ultra-Wide Field Fluorescein Angiography: DAVE Study. Retina.

[64] Fan W., Nittala M.G., Fleming A., Robertson G., Uji A., Wykoff C.C., Brown D.M., van Hemert J., Ip M., Wang K. (2020). Relationship Between Retinal Fractal Dimension and Nonperfusion in Diabetic Retinopathy on Ultrawide-Field Fluorescein Angiography. Am. J. Ophthalmol..

[65] Sevgi D.D., Srivastava S.K., Whitney J., O’Connell M., Kar S.S., Hu M., Reese J., Madabhushi A., Ehlers J.P. (2021). Characterization of Ultra-Widefield Angiographic Vascular Features in Diabetic Retinopathy with Automated Severity Classification. Ophthalmol. Sci..

[66] Fang M., Fan W., Shi Y., Ip M.S., Wykoff C.C., Wang K., Falavarjani K.G., Brown D.M., van Hemert J., Sadda S.R. (2019). Classification of Regions of Nonperfusion on Ultra-widefield Fluorescein Angiography in Patients with Diabetic Macular Edema. Am. J. Ophthalmol..

[67] Moosavi A., Figueiredo N., Prasanna P., Srivastava S.K., Sharma S., Madabhushi A., Ehlers J.P. (2021). Imaging Features of Vessels and Leakage Patterns Predict Extended Interval Aflibercept Dosing Using Ultra-Widefield Angiography in Retinal Vascular Disease: Findings From the PERMEATE Study. IEEE Trans. Biomed. Eng..

[68] Hormel T.T., Jia Y., Jian Y., Hwang T.S., Bailey S.T., Pennesi M.E., Wilson D.J., Morrison J.C., Huang D. (2021). Plexus-specific retinal vascular anatomy and pathologies as seen by projection-resolved optical coherence tomographic angiography. Prog. Retin. Eye Res..

[69] Shahlaee A., Pefkianaki M., Hsu J., Ho A.C. (2016). Measurement of Foveal Avascular Zone Dimensions and its Reliability in Healthy Eyes Using Optical Coherence Tomography Angiography. Am. J. Ophthalmol..

[70] Barraso M., Alé-Chilet A., Hernández T., Oliva C., Vinagre I., Ortega E., Figueras-Roca M., Sala-Puigdollers A., Esquinas C., Esmatjes E. (2020). Optical Coherence Tomography Angiography in Type 1 Diabetes Mellitus. Report 1: Diabetic Retinopathy. Transl. Vis. Sci. Technol..

[71] Salz D.A., De Carlo T.E., Adhi M., Moult E.M., Choi W., Baumal C.R., Witkin A.J., Duker J.S., Fujimoto J.G., Waheed N.K. (2016). Select Features of Diabetic Retinopathy on Swept-Source Optical Coherence Tomographic Angiography Compared with Fluorescein Angiography and Normal Eyes. JAMA Ophthalmol..

[72] Freiberg F.J., Pfau M., Wons J., Wirth M.A., Becker M.D., Michels S. (2016). Optical coherence tomography angiography of the foveal avascular zone in diabetic retinopathy. Graefe’s Arch. Clin. Exp. Ophthalmol..

[73] Balaratnasingam C., Inoue M., Ahn S., McCann J., Dhrami-Gavazi E., Yannuzzi L.A., Freund K.B. (2016). Visual Acuity Is Correlated with the Area of the Foveal Avascular Zone in Diabetic Retinopathy and Retinal Vein Occlusion. Ophthalmology.

[74] Samara W.A., Shahlaee A., Adam M., Khan M.A., Chiang A., Maguire J.I., Hsu J., Ho A.C. (2017). Quantification of Diabetic Macular Ischemia Using Optical Coherence Tomography Angiography and Its Relationship with Visual Acuity. Ophthalmology.

[75] Lee H., Lee M., Chung H., Kim H.C. (2018). Quantification Of Retinal Vessel Tortuosity In Diabetic Retinopathy Using Optical Coherence Tomography Angiography. Retina.

[76] Zarranz-Ventura J., Barraso M., Alé-Chilet A., Hernandez T., Oliva C., Gascón J., Sala-Puigdollers A., Figueras-Roca M., Vinagre I., Ortega E. (2019). Evaluation of microvascular changes in the perifoveal vascular network using optical coherence tomography angiography (OCTA) in type I diabetes mellitus: A large scale prospective trial. BMC Med. Imaging.

[77] Chu Z., Lin J., Gao C., Xin C., Zhang Q., Chen C.-L., Roisman L., Gregori G., Rosenfeld P.J., Wang R. (2016). Quantitative assessment of the retinal microvasculature using optical coherence tomography angiography. J. Biomed. Opt..

[78] Dupas B., Minvielle W., Bonnin S., Couturier A., Erginay A., Massin P., Gaudric A., Tadayoni R. (2018). Association Between Vessel Density and Visual Acuity in Patients with Diabetic Retinopathy and Poorly Controlled Type 1 Diabetes. JAMA Ophthalmol..

[79] Nguyen T.T., Wang J.J., Sharrett A.R., Islam F.A., Klein R., Klein B.E., Cotch M.F., Wong T.Y. (2007). Relationship of Retinal Vascular Caliber with Diabetes and Retinopathy: The Multi-Ethnic Study of Atherosclerosis (MESA). Diabetes Care.

[80] Tsai A.S., Wong T.Y., Lavanya R., Zhang R., Hamzah H., Tai E.S., Cheung C. (2011). Differential association of retinal arteriolar and venular caliber with diabetes and retinopathy. Diabetes Res. Clin. Pr..

[81] Tang F.Y., Ng D.S., Lam A., Luk F., Wong R., Chan C., Mohamed S., Fong A., Lok J., Tso T. (2017). Determinants of Quantitative Optical Coherence Tomography Angiography Metrics in Patients with Diabetes. Sci. Rep..

[82] Maloca P.M., Spaide R.F., De Carvalho E.R., Studer H.P., Hasler P.W., Scholl H.P.N., Heeren T., Schottenhamml J., Balaskas K., IOB Study Group (2020). Novel biomarker of sphericity and cylindricity indices in volume-rendering optical coherence tomography angiography in normal and diabetic eyes: A preliminary study. Graefe’s Arch. Clin. Exp. Ophthalmol..

[83] Le D., Alam M., Miao B.A., Lim J.I., Yao X. (2019). Fully automated geometric feature analysis in optical coherence tomography angiography for objective classification of diabetic retinopathy. Biomed. Opt. Express.

[84] Nassisi M., Lei J., Abdelfattah N., Karamat A., Balasubramanian S., Fan W., Uji A., Marion K.M., Baker K., Huang X. (2019). OCT Risk Factors for Development of Late Age-Related Macular Degeneration in the Fellow Eyes of Patients Enrolled in the HARBOR Study. Ophthalmology.

[85] Toth C.A., Tai V., Chiu S.J., Winter K., Sevilla M.B., Daniel E., Grunwald J.E., Jaffe G.J., Martin D.F., Ying G.-S. (2017). Linking OCT, Angiographic, and Photographic Lesion Components in Neovascular Age-Related Macular Degeneration. Ophthalmol. Retin..

[86] Lunasco L., Abraham J.R., Sarici K., Sevgi D.D., Hanumanthu A., Cetin H., Hu M., Srivastava S., Reese J., Ehlers J.P. (2021). Comparative Assessment of Long-Term Longitudinal Multi-Layer Retinal Dynamics in Non-neovascular Age-Related Macular Degeneration in Eyes Progressing to Subfoveal Geographic Atrophy and Eyes without Progression. Investig. Ophthalmol. Vis. Sci..

[87] Hanumanthu A., Sarici K., Abraham J.R., Whitney J., Lunasco L., Sevgi D.D., Cetin H., Srivastava S.K., Reese J., Ehlers J.P. (2021). Utilizing Higher-Order Quantitative SD-OCT Biomarkers in a Machine Learning Prediction Model for the Development of Subfoveal Geographic Atrophy in Age-Related Macular Degeneration. Investig. Ophthalmol. Vis. Sci..

[88] Lunasco L., Abraham J.R., Sarici K., Sevgi D.D., Hanumanthu A., Cetin H., Hu M., Srivastava S.K., Reese J., Ehlers J.P. (2021). Risk Classification for Progression to Subfoveal Geographic Atrophy in Dry Age-Related Macular Degeneration Using Machine Learning-Enabled Outer Retinal Feature Extraction. OSLI Retin..

[89] Abdelfattah N., Zhang H., Boyer D.S., Rosenfeld P.J., Feuer W.J., Gregori G., Sadda S.R. (2016). Drusen Volume as a Predictor of Disease Progression in Patients with Late Age-Related Macular Degeneration in the Fellow Eye. Investig. Opthalmol. Vis. Sci..

[90] Ehlers J.P., Zahid R., Kaiser P.K., Heier J.S., Brown D.M., Meng X., Reese J., Le T.K., Lunasco L., Hu M. (2021). Longitudinal Assessment of Ellipsoid Zone Integrity, Subretinal Hyperreflective Material, and Subretinal Pigment Epithelium Disease in Neovascular Age-Related Macular Degeneration. Ophthalmol. Retin..

[91] Waldstein S., Philip A.-M., Leitner R., Simader C., Langs G., Gerendas B.S., Schmidt-Erfurth U. (2016). Correlation of 3-Dimensionally Quantified Intraretinal and Subretinal Fluid with Visual Acuity in Neovascular Age-Related Macular Degeneration. JAMA Ophthalmol..

[92] De Fauw J., Ledsam J.R., Romera-Paredes B., Nikolov S., Tomasev N., Blackwell S., Askham H., Glorot X., O’Donoghue B., Visentin D. (2018). Clinically applicable deep learning for diagnosis and referral in retinal disease. Nat. Med..

[93] Lee C.S., Baughman D.M., Lee A.Y. (2017). Deep Learning Is Effective for Classifying Normal versus Age-Related Macular Degeneration OCT Images. Ophthalmol. Retin..

[94] De Sisternes L., Simon N., Tibshirani R., Leng T., Rubin D.L. (2014). Quantitative SD-OCT Imaging Biomarkers as Indicators of Age-Related Macular Degeneration Progression. Investig. Opthalmology Vis. Sci..

[95] Schmidt-Erfurth U., Waldstein S.M., Klimscha S., Sadeghipour A., Hu X., Gerendas B.S., Osborne A., Bogunović H. (2018). Prediction of Individual Disease Conversion in Early AMD Using Artificial Intelligence. Investig. Ophthalmol. Vis. Sci..

[96] Wong W.L., Su X., Li X., Cheung C.M.G., Klein R., Cheng C.Y., Wong T.Y. (2014). Global prevalence of age-related macular degeneration and disease burden projection for 2020 and 2040: A systematic review and meta-analysis. Lancet Glob. Health.

[97] Freund K.B., Yannuzzi L.A., Sorenson J.A. (1993). Age-related Macular Degeneration and Choroidal Neovascularization. Am. J. Ophthalmol..

[98] Jia Y., Bailey S.T., Hwang T.S., McClintic S.M., Gao S.S., Pennesi M.E., Flaxel C.J., Lauer A.K., Wilson D.J., Hornegger J. (2015). Quantitative optical coherence tomography angiography of vascular abnormalities in the living human eye. Proc. Natl. Acad. Sci. USA.

[99] Spaide R.F., Klancnik J.M., Cooney M.J. (2015). Retinal Vascular Layers Imaged by Fluorescein Angiography and Optical Coherence Tomography Angiography. JAMA Ophthalmol..

[100] Cicinelli M.V., Rabiolo A., Sacconi R., Carnevali A., Querques L., Bandello F., Querques G. (2018). Optical coherence tomography angiography in dry age-related macular degeneration. Surv. Ophthalmol..

[101] Jia Y., Bailey S.T., Wilson D.J., Tan O., Klein M.L., Flaxel C.J., Potsaid B., Liu J.J., Lu C.D., Kraus M.F. (2014). Quantitative Optical Coherence Tomography Angiography of Choroidal Neovascularization in Age-Related Macular Degeneration. Ophthalmology.

[102] Uchida A., Hu M., Babiuch A., Srivastava S.K., Singh R.P., Kaiser P.K., Talcott K., Rachitskaya A., Ehlers J.P. (2019). Optical coherence tomography angiography characteristics of choroidal neovascularization requiring varied dosing frequencies in treat-and-extend management: An analysis of the AVATAR study. PLoS ONE.

[103] Chatziralli I., Theodossiadis G., Panagiotidis D., Pousoulidi P., Theodossiadis P. (2018). Choriocapillaris Vascular Density Changes in Patients with Drusen: Cross-Sectional Study Based on Optical Coherence Tomography Angiography Findings. Ophthalmol. Ther..

[104] Lane M., Moult E.M., Novais E.A., Louzada R.N., Cole E.D., Lee B., Husvogt L., Keane P.A., Denniston A.K., Witkin A.J. (2016). Visualizing the Choriocapillaris Under Drusen: Comparing 1050-nm Swept-Source Versus 840-nm Spectral-Domain Optical Coherence Tomography Angiography. Investig. Opthalmol. Vis. Sci..

[105] Byon I., Ji Y., Alagorie A.R., Tiosano L., Sadda S.R. (2021). Topographic Assessment Of Choriocapillaris Flow Deficits In The Intermediate Age-Related Macular Degeneration Eyes With Hyporeflective Cores Inside Drusen. Retina.

[106] Choi W., Moult E.M., Waheed N.K., Adhi M., Lee B., Lu C.D., de Carlo T.E., Jayaraman V., Rosenfeld P.J., Duker J.S. (2015). Ultrahigh-Speed, Swept-Source Optical Coherence Tomography Angiography in Nonexudative Age-Related Macular Degeneration with Geographic Atrophy. Ophthalmology.

[107] Camino A., Guo Y., You Q.S., Wang J., Huang D., Bailey S.T., Jia Y. (2019). Detecting and measuring areas of choriocapillaris low perfusion in intermediate, non-neovascular age-related macular degeneration. Neurophotonics.

